# A Role for the RNA Chaperone Hfq in Controlling Adherent-Invasive *Escherichia coli* Colonization and Virulence

**DOI:** 10.1371/journal.pone.0016387

**Published:** 2011-01-26

**Authors:** Karina T. Simonsen, Gorm Nielsen, Janni Vester Bjerrum, Thomas Kruse, Birgitte H. Kallipolitis, Jakob Møller-Jensen

**Affiliations:** 1 Department of Biochemistry and Molecular Biology, University of Southern Denmark, Odense, Denmark; 2 Novozymes, Bagsværd, Denmark; Baylor College of Medicine, United States of America

## Abstract

Adherent-invasive *Escherichia coli* (AIEC) has been linked with the onset and perpetuation of inflammatory bowel diseases. The AIEC strain LF82 was originally isolated from an ileal biopsy from a patient with Crohn's disease. The pathogenesis of LF82 results from its abnormal adherence to and subsequent invasion of the intestinal epithelium coupled with its ability to survive phagocytosis by macrophages once it has crossed the intestinal barrier. To gain further insight into AIEC pathogenesis we employed the nematode *Caenorhabditis elegans* as an *in vivo* infection model. We demonstrate that AIEC strain LF82 forms a persistent infection in *C. elegans*, thereby reducing the host lifespan significantly. This host killing phenotype was associated with massive bacterial colonization of the nematode intestine and damage to the intestinal epithelial surface. *C. elegans* killing was independent of known LF82 virulence determinants but was abolished by deletion of the LF82 *hfq* gene, which encodes an RNA chaperone involved in mediating posttranscriptional gene regulation by small non-coding RNAs. This finding reveals that important aspects of LF82 pathogenesis are controlled at the posttranscriptional level by riboregulation. The role of Hfq in LF82 virulence was independent of its function in regulating RpoS and RpoE activity. Further, LF82Δ*hfq* mutants were non-motile, impaired in cell invasion and highly sensitive to various chemical stress conditions, reinforcing the multifaceted function of Hfq in mediating bacterial adaptation. This study highlights the usefulness of simple non-mammalian infection systems for the identification and analysis of bacterial virulence factors.

## Introduction

*Escherichia coli* is normally found as a harmless commensal colonizing the mucous layer of the mammalian colon. However, a number of pathogenic *E. coli* strains have adapted to other niches, causing diverse intestinal and extraintestinal diseases [1], [2]. A relatively unexplored pathotype is the adherent-invasive *E. coli* (AIEC), which was first isolated from the ileal mucosa of a patient with Crohn's disease (CD) [3]. CD is a chronic relapsing inflammatory bowel disease for which the exact etiology is still unknown. The uncontrolled inflammation of the intestine characteristic of CD appears to arise by a complex interplay between changes in the composition of the enteric mucosal microbiota (termed dysbiosis) on the one hand, and dysregulation of the mucosal immune system on the other. The specific overrepresentation of certain bacterial species in the intestinal mucosa may indeed serve as the trigger that elicits pathological responses in genetically susceptible individuals with reduced microbial clearance [4]. Numerous independent studies have reported the specific overrepresentation of AIEC species in biopsies from patients with CD, ulcerative colitis and colon cancer, thus pointing to this pathotype as an important culprit in determining the onset and perpetuation of inflammatory bowel diseases [5]–[13].

AIEC are defined according to the characteristics of the prototypical strain LF82, i.e. (i) the ability to adhere to and invade intestinal epithelial cells, (ii) the ability to persist and multiply intracellularly in epithelial cells after lysis of the endocytic vacuole, and (iii) the capacity to survive and replicate *inside* maturing phagolysosomes of cultured marcophages and induce the release of TNF-α [7], [14]. Adhesion of LF82 requires the expression of type I pili which bind to the carcinoembryonic antigen-related cell adhesion molecule 6 (CEACAM6) present in high amounts on the brush border of enterocytes from CD patients [15], [16]. Apart from mediating cell adhesion, type I pili play a role in promoting cell invasion by a macropinocytosis-like process which involves both the host cell actin filaments and microtubules [17]. LF82 does not contain any of the known *E. coli* invasive determinants, such as *ipaC* of enteroinvasive *Escherichia coli*, *eae* of enteropathogenic *Escherichia coli*, and *tia* of enterotoxigenic *Escherichia coli*, and the exact mechanism of invasion remains to be determined [15]–[17].

So far, the pathogenicity of AIEC has been studied mainly by use of cultured cell lines. AIEC efficiently invades a wide range of epithelial cell lines including Hep-2 cells and the intestinal cell lines Intestine-407, Caco-2 and HCT-8 [17]. Infection studies using cell culture have led to the identification of major virulence factors including type 1 pili [16], outer membrane vesicles (OMV) that may serve as delivery vehicles for effector molecules to host cells [18], and outer membrane protein C (OmpC) which affects AIEC adhesion and invasion indirectly via the sigma(E) regulatory pathway [19]. Likewise, genes that are important for AIEC survival and extensive intracellular multiplication have been identified by use of cultured murine macrophages. By contrast to most invasive bacteria, which either induce death of infected macrophages or escape the normal endocytic pathway, AIEC remain present within maturing phagosomes, thus resisting the acidic pH, oxidative stress, proteolytic activity and antimicrobial compounds found inside the phagocytic vacuole [20]. Screening of an LF82 transposon mutant library for attenuated ability to resist macrophage killing led to the identification of the important virulence determinants *htrA* and *dsbA* [21], [22]. The bacterial stress protein HtrA (high-temperature requirement A) has been found to be induced in intramacrophagic AIEC bacteria and to be essential for bacterial intracellular multiplication [21]. DsbA, a periplasmic oxidoreductase, has been found to be required both for bacterial adherence to epithelial cells, through its effect on type 1 pilus expression, and for bacterial resistance to macrophage killing [22]. A mouse model of colonic inflammation was developed recently for the study of AIEC pathogenicity *in vivo* [23]. In contrast to nonpathogenic *E. coli* K12, virulent LF82 bacteria exacerbated the induced mouse colonic inflammation by potentiating the inflammatory mucosal immune response in a manner dependent on the bacterial flagellum [23]. In addition, transgenic mice expressing the human CEACAM proteins have been used to confirm the importance of bacterial type 1 pili for colonization of the intestinal mucosa and induction of gut inflammation [24].

The soil nematode *Caenorhabditis elegans* has been employed in numerous recent studies as a simple animal model for the study of host-pathogen interactions, generating important insights into both bacterial pathogenesis and host innate immunity [25], [26]. Many of the virulence mechanisms used by bacterial pathogens to cause disease in mammalian hosts have also been shown to be important for pathogenesis in *C. elegans* and, similarly, important features of the host innate immunity have been evolutionarily conserved between *C. elegans* and mammals. Several important human pathogens, including *Pseudomonas aeruginosa* [27], *Salmonella enterica* [28], [29], *Staphylococcus aureus* [30], and *E. coli* [31], have been investigated using *C. elegans*. At least two distinct mechanisms exist by which bacterial pathogens kill *C. elegans*: “slow killing” which takes place over the course of several days and is associated with bacterial infection of the nematode, and “fast killing” which is mediated by diffusible toxins and is not associated with the accumulation of bacteria in the nematode intestine [26].

In attempt to identify additional AIEC virulence factors, we chose to investigate LF82 infection using *C. elegans* as a host organism. Here we report the establishment of *C. elegans* as a useful model system for LF82 infection. Persistent colonization by LF82 results in a robust slow-killing phenotype, which is independent of known LF82 virulence determinants. LF82 virulence was found to be strictly dependent on the RNA-binding protein Hfq, which also plays important roles in bacterial stress tolerance and motility. The fact that Hfq was found also to be important for epithelial cell invasion and intracellular survival in cultured macrophages points to a central role of Hfq in orchestrating LF82 virulence in distinct infection niches.

## Materials and Methods

### Bacterial and nematode strains, plasmids, and growth conditions

The *Escherichia coli* strains, plasmids and PCR primers used are listed in Table S1. *E. coli* DH5α was used for standard cloning procedures. Mutant strains were constructed in the BW25113 strain using the lambda Red-mediated recombination as described previously [32]. Antibiotics resistance cassettes were subsequently transferred into the ampicillin sensitive LF82 derivate LF82* (referred to as LF82 throughout) by P1 phage transduction. The *E. coli hfq* coding region was PCR amplified using the primers JMJ153 and JMJ154 and cloned into the IPTG-inducible expression vector pRBJ200 using *BamHI* and *EcoRI* restriction enzymes, thereby creating pJMJ220. The empty vector pNDM220 served as negative control in complementation experiments. A kanamycin resistance variant of pEGFP, pEGFPk was constructed by inserting an *aphA*-containg *Stu*I restriction fragment from pACYC177 into pEGFP.

*E. coli* strains were routinely cultured in LB-medium at 37°C or 30°C with shaking. For virulence experiments, LF82 and mutant derivates were cultured without agitation in order to induce virulence determinants [17].

*C. elegans* strain SS104 [*glp-4*(bn2)I] was obtained from the *Caenorhabditis* Genetics Center and maintained at 15°C on NGM (nematode growth medium) agar with *E*. *coli* strain OP50 using established procedures (Brenner, 1974). Killing assays and colonization assays were performed on NGM plates with the following additions where appropriate: nystatin 50 µg/ml, kanamycin at 50 µg/ml, ampicillin at 30 µg/ml and IPTG at 1 mM.

### *C. elegans* killing assay

To avoid confounding effects of progeny in the killing assays, we used the worm strain *glp-4*(*bn2*) since this temperature sensitive mutants is sterile at 25°C. L1 larvae synchronized by hypochlorite treatment and overnight starvation in M9 buffer were placed onto OP50-seeded NGM plates and grown at the restrictive temperature (25°C). L4 or young adults were washed three times with M9 buffer and transferred to the killing plates. These plates were prepared by seeding NGM plates containing the antifungal compound nystatin and appropriate antibiotics with fresh LF82 or OP50 (control) overnight cultures concentrated 10x in fresh LB media. The plates incubated overnight at 37°C and cooled to room temperature before 30–50 animals were transferred to each plate. Plates were incubated at 25°C and scored for live worms daily. Worms were considered dead and removed when they did not respond to gentle prodding with a platinum wire. Survival data from two plates were compiled in order to produce Kaplan-Meier survival curves. Statistical analysis using the logrank test in the GraphPad Prism 4.03 software (GraphPad Prism Software, Inc) was performed and a *P* value of <0.05 was considered statistically significant. All killing assays were performed at least twice with similar results.

### Nematode colonization measurement

The bacterial load in the nematode intestine was determined essentially as described previously [33]. 10 infected nematodes were transferred to a microcentrifuge tube with 1 ml of M9 buffer containing 1 mM sodium azide to inhibit expulsion of bacteria from the nematode intestine. Nematodes were washed three times by removing 750 µl of buffer and restoring the volume to 1 ml. A 10-µl sample was removed from the resulting 250-µl nematode suspension, diluted 100-fold and plated to determine the number of external colony-forming units (CFU). Approximately 400 mg of 1.0-mm silicon carbide particles (Catalog no. 11079110sc; Biospec Products, Bartlesville, OK) was then added to each tube. Tubes were then vortexed at maximum speed for one minute to disrupt the nematodes while leaving the bacteria unaffected. The resulting suspension was diluted and plated onto selective media to determine CFU. Measurements were performed at least two times in duplicate.

### Assay of infection persistency

Synchronized L4 nematodes and 5-cm NGM plates seeded with bacteria were prepared as described above. 30–50 L4 nematodes were transferred to preformed LF82/pEGFPk lawns and allowed to feed for 24 hours and removed. In order to reduce LF82 carryover, nematodes were washed twice in 500 µl of M9 buffer placed in glass depression slides before placing on OP50/pQW58 lawns. After that, worm killing assays were performed as described above and the presence of LF82/pEGFP was determined by CFU-determination on selective kanamycin agar plates and fluorescence microscopy using a Zeiss-LSM 510 META NLO inverted microscope (Zeiss, Jena, Germany).

### Cell lines and cell culture

J774-A1, a murine macrophage-like cell line (ATCC TIB-67) was maintained in a 5% CO_2_ atmosphere at 37°C in RPMI 1640 medium (Biowhittaker) supplemented with 2.05 mM L-glutamine, 10% heat-inactivated fetal calf serum (FCS) and 50 µg/ml gentamicin. HeLaS3 cells (ATCC CCL-2.2) were cultured at 37°C in 5% CO_2_ in Dulbecco's modified Eagle's medium (DMEM; Gibco) supplemented 4.5 g/L glucose, 4 mM L-glutamine, 110 mg/ml pyruvate, 10% heat-inactivated FCS and 50 µg/ml gentamicin.

### Cell culture invasion and intracellular multiplication

Bacterial invasion of epithelial cells was determined as protection against the bacteriocidal antibiotic gentamicin as described previously [34]. Hela cells were collected by trypsination and seeded in 12-well tissue culture plates with approximately 2×10^5^ cells per well and allowed to grow overnight in 2 ml DMEM medium +10% FCS +50 µg/ml gentamicin at 37°C in a 5% CO_2_ atmosphere. Cells were washed three times in prewarmed PBS before addition of 2 ml of prewarmed DMEM +10% FCS without gentamicin. Bacterial cultures were grown overnight in LB medium containing antibiotics without agitation and washed in 0.9% NaCl before infection. Each monolayer was infected at a multiplicity of infection (MOI) of 100 bacteria per epithelial cell. The tissue culture plates were centrifuged in a prewarmed centrifuge at 200×g for 5 minutes to promote bacterial adherence to the cell monolayer and subsequently incubated at 37°C in a 5% CO_2_ atmosphere. After a 2-hour infection period the monolayers were washed three times in prewarmed PBS before addition of 2 ml prewarmed DMEM +10% FCS +100 µg/ml gentamicin to kill extracellular bacteria. After 1 hour incubation at 37°C in a 5% CO_2_ atmosphere cells were washed three times in PBS and lysed by addition of 1 mL 0.1% Triton X-100 for five minutes. Samples were removed, diluted in 0.9% NaCl, and plated on agar plates to determine the number of CFU recovered from the lysed cell monolayers. A measure of the relative invasiveness of the LF82Δ*hfq* mutant was obtained by relating the CFU number to that resulting from infection with the wild type strain (invasion index  = 100). All measurements were conducted at least two times in triplicate.

### Intracellular survival and replication in macrophages

The capacity of LF82 and mutant strains to survive and replicate intracellularly in macrophages was measured by gentamicin protection as described above. J774-A1 cells were collected by trypsination and seeded in 12-well plates at a density of 5×10^5^ cells per well and incubated for 24 hours in RPMI 1640 medium +10% FCS +50 µg/ml gentamicin at 37°C in a 5% CO_2_ atmosphere. Prior to infection the cell monolayer was washed three times in prewarmed PBS. Macrophage monolayers were infected at a multiplicity of infection (MOI) of 100 bacteria per cell in 2 ml of RPMI medium without gentamicin, centrifuged at 200×g for 5 minutes, and incubated for 1 hour at 37°C in a 5% CO_2_ atmosphere. After infection the cells were washed three times with PBS before addition of prewarmed medium containing 100 µg/ml gentamicin for 1 hour. Cells used for 2-hour samples were washed three times in PBS and lysed by addition of 0.1% Triton X-100 followed by dilution and plating to determine intracellular CFU. Infected cells used for 24-hour samples were incubated overnight in RPMI 1640 medium +10% FCS +10 µg/ml gentamicin at 37°C in a 5% CO_2_ atmosphere before lysis and plating. Intracellular survival and replication was determined as fraction of the bacterial input. All measurements were performed at least two times in duplicate.

### Bacterial stress tolerance test

Stress tolerance tests were carried out as described [35]. Bacterial cultures were grown at 37°C in LB medium to an OD_600_ = 0.1 and diluted in ten-fold steps in 0.9% NaCl. 5 µl samples were spotted onto LB agar plates containing 1 mM IPTG, 30 µl/ml ampicillin when appropriate, and one of the following stress agents: 100 mM MES pH 5.0, 100 mM MES pH 5.0 + 1 mM NaNO_2_, 1 mM methyl viologen, 1 mM H_2_O_2_. Plates were incubated overnight at 37°C. Stress tests were performed three times and single representative results are presented.

### Transmission electron microscopy

For negative stain electron microscopy, bacteria grown overnight in LB broth at 37°C without agitation were placed for 1 minute on carbon-formvar copper grids (FCF-200-Cu; Electron Microscopy Sciences, UK) and negatively stained for 1 minute with 0.5% phosphotungstic acid, pH 6.0 (Sigma). For electron microscopy of thin sections, a synchronized worm population was propagated on NGM plates containing different bacterial strains as described for the killing assay above. Worms were washed off NGM plates with sterile H_2_O, rinsed three times in sterile H_2_O and fixed in 1 mL 2% glutaraldehyde in 0.04 M phosphate buffer for 24 hours at 4°C. Following centrifugation at 200 rpm for five minutes, the worms were washed in 0.1 M phosphate buffer and post-fixed in 0.5% OsO_4_ and 0.5% KFe(CN)_6_ in 0.1 M cacodylate for 90 minutes on ice. This was followed by three washes in 0.1 M cacodylate, and three washes in 0.1 M sodium acetate. Fixed worms were stained in 1% uranyl acetate in 0.1 M sodium acetate (pH 5.2) for one hour at room temperature, and washed three times in 0.1 M sodium acetate and three times in water. 15% BSA was added, and samples were incubated for one hour at room temperature. The worms were collected by centrifugation and fixed in 2% glutaraldehyde in 0.04 M phosphate buffer over night at 4°C. Samples were dehydrated through a graded series of ethanol to 100%, two times 15 minutes in 100% propylene oxide, then 30 minutes in propylene oxide/EPON (1∶1), 30 minutes in propylene oxide/EPON (1∶3), and overnight in EPON. 50-nm sections of specimen were cut on a Leica Ultra-cut microtome and transferred onto formvar-coated cupper grids. Grids were stained in 3% uranyl acetate for 14 minutes at 60°C, rinsed with sterile H_2_O, stained with lead citrate for 6 minutes at room temperature and finally rinsed with sterile H_2_O containing a few drops of concentrated NaOH to remove excess stain. Transmission electron microscopy was performed at 80 kV using a Philips EM208 electron microscope.

### Motility assays

LF82 and the LF82Δ*hfq* mutant strain were grown statically in LB broth and adjusted to similar densities by addition of prewarmed medium to the LF82 culture. In order to measure motility, 2-µl samples were spotted onto swimming agar plates (0.3% Difco agar, 0.4% glucose in LB broth) or swarming agar plates (0.45% Eiken agar, 0.4% glucose in LB broth), and incubated for 8 and 18 hours, respectively before inspection. Agar plates were allowed to dry overnight at room temperature and preheated to 37°C before use.

### MIC determinations

MIC (minimal inhibitory concentration) assays were performed according to CSLI guidelines except that cells were grown in 1/5 Mueller Hinton Broth. All antimicrobial peptides were produced by solid phase chemical synthesis and provided by the following suppliers: Cathelicidin LL-37, novicidin, iseganan and omiganan (Ross-Petersen Aps); magainin I and cecropin A (Sigma); hBD2 and HD5 (Peptide Institute INC.). Polymyxin B and lysozyme were purchased from Sigma.

### Yeast agglutination assay

5 ml cultures of bacterial strains were grown overnight at 37°C without shaking. Agglutination was assayed on glass slides by mixing 15 µl bacterial culture with an equal volume of bakers' yeast suspension (10 mg/ml). Mannose inhibition of agglutination was confirmed by addition of 3% α-D-mannose to the bacteria prior to mixing with the yeast suspension.

## Results

### Adherent-invasive *Escherichia coli* LF82 establishes a persistent colonization in *C. elegans* that kills the host

**Figure 1:**
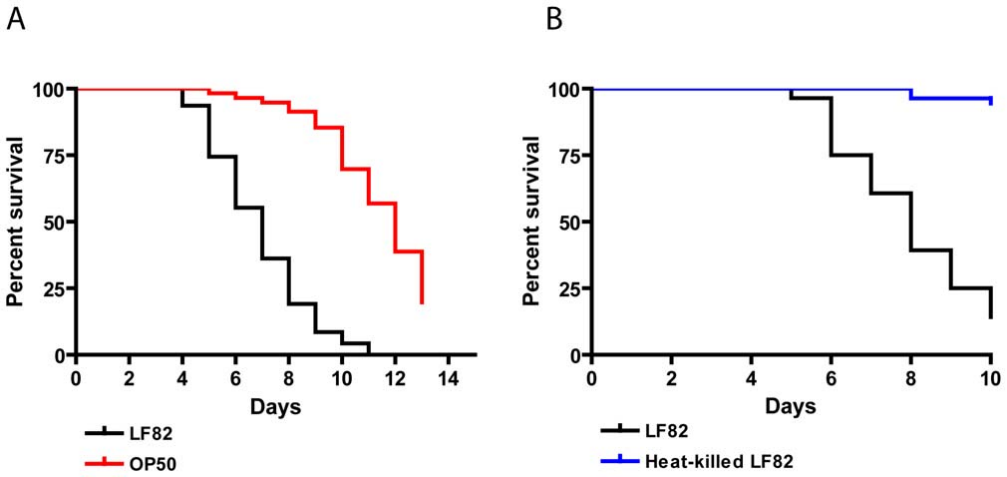
LF82 kills the *C. elegans* nematode. (**A**) Nematode killing assay. Synchronized populations of *C. elegans glp-4* nematodes were propagated on standard substrate (*E. coli* OP50) to the L4 larval stage and transferred to lawns of adherent-invasive *E. coli* LF82 (worm population size n = 47) and non-pathogenic *E. coli* OP50 (n = 116), respectively, grown on NGM medium. The *C. elegans* life span was significantly reduced when feeding on LF82 compared to the OP50 control (P<0.0001). (**B**) Killing assay comparing nematodes feeding on live (n = 26) and heat-killed LF82 (n = 52) (P<0.0001).

In order to determine the suitability of *C. elegans* as a model system for studying LF82 pathogenesis, we tested this *E. coli* strain for its ability to kill *C. elegans* when the worms feed on a lawn of LF82 on NGM plates. As feeding on LF82 did not prevent the formation of nematode progeny (data not shown), we used the temperature-sensitive germ-line mutant *glp-4* in order to ensure a defined number of age synchronized nematodes for the duration of the killing experiments. As shown in Figure 1A, LF82 displayed a robust virulence phenotype significantly reducing the nematode life span compared to the non-virulent OP50 control (*P*<0.0001). *C. elegans* feeding on LF82 displayed 50% mortality (LT_50_) in 6.3 days compared to 11.3 days for nematodes feeding on OP50. None of the nematodes died within the first three days of the experiment, indicating that LF82 killing takes place via a ‘slow killing’ mechanism resulting from bacterial infection of the host rather than ‘fast killing’ mediated by a diffusible toxin [25]. In support hereof, LF82 displayed no killing phenotype when heated to 65°C for 30 minutes before being spread on NGM plates (Figure 1B).

**Figure 2:**
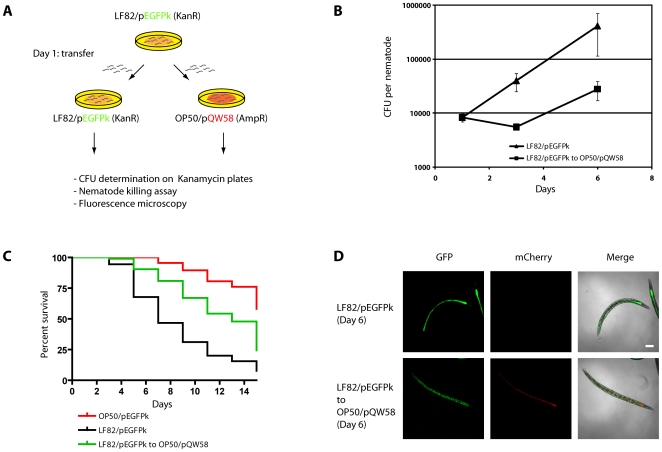
*C. elegans* killing by LF82 results from a persistent colonization. (**A**) Assay of colonization persistency: *C. elegans* propagated on LF82/pEGFPk for 24 hours were transferred to LF82/pEGFPk or OP50/pQW58, respectively. At subsequent time points, nematodes were analyzed for bacterial colonization by (**B**) plating nematode homogenates on kanamycin plates for LF82 CFU measurement, (**C**) monitoration of nematode survival in killing assays (OP50/pEGFPk (n = 67); LF82/pEGFPk (n = 183); LF82/pEGFPk to OP50/pQW58 (n = 94), and (**D**) fluorescence microscopic examination of nematodes for the presence of GFP- and mCherry-expressing bacteria in the nematode intestinal lumen on day 6 post infection. Nematode survival when transferred from LF82/pEGFPk to OP50/pQW58 is significantly different from that of nematodes feeding only on LF82/pEGFPk(P<0.0001) and OP50/pEGFPk (P<0.0001).

Slow killing of *C. elegans* has been observed for other infecting bacteria including *Pseudomonas aeruginosa* [27] and *Salmonella typhimurium* [28], [29]. In contrast to *P. aeruginosa*, *S. typhimurium* are capable of persisting within the nematode gut after transfer of the host onto a lawn of *E. coli*. To investigate if the LF82 killing phenotype reflects a persistent bacterial infection, we conducted a nematode transfer experiment outlined in Figure 2A. A synchronized population of *C. elegans* was allowed to feed on LF82/pEGFPk bacteria for 24 hours before being transferred onto lawns of either LF82/pEGFPk or OP50/pQW58. The ability of LF82/pEGFPk to persist within the nematodes was subsequently investigated at various time points after transfer by CFU determination (Figure 2B), host killing assay (Figure 2C), and fluorescence microscopy (Figure 2D). Nematode transfer onto plates containing OP50/pQW58 did not eliminate LF82/pEGFPk from the nematode intestine and by day 6 after infection the remaining fraction had in fact increased in number, suggesting that LF82 is capable of persisting and multiplying inside the living host (Figure 2B, squares). The persistent colonization by LF82 was correlated with shortening of the host life-span, as determined in the killing assay (Figure 2C). Expression of GFP and the red-fluorescent mCherry proteins by LF82/pEGFPk and OP50/pQW58, respectively, allowed for fluorescence-microscopic examination of the infected nematodes. At day 5 after transfer, nematodes were inspected for the presence of LF82/pEGFPk and OP50/pQW58. Figure 2D shows that the green fluorescence is located in the intestinal lumen of the host. The GFP fluorescence level is lower in nematodes transferred to OP50/pQW58 after 24 hours, consistent with the reduced CFU count obtained for these nematodes. In addition, red-fluorescent OP50/QW58 appears in the nematode intestinal lumen. This finding most likely reflects the weakened condition of the nematodes since OP50 colonization is not observed at this stage in healthy nematodes fed only on OP50 (data not shown). Thus, we conclude that adherent-invasive *E. coli* LF82 is able to establish a persistent infection in *C. elegans* which ultimately kills the host.

### The LF82 *hfq* gene is required for nematode colonization and killing

**Figure 3:**
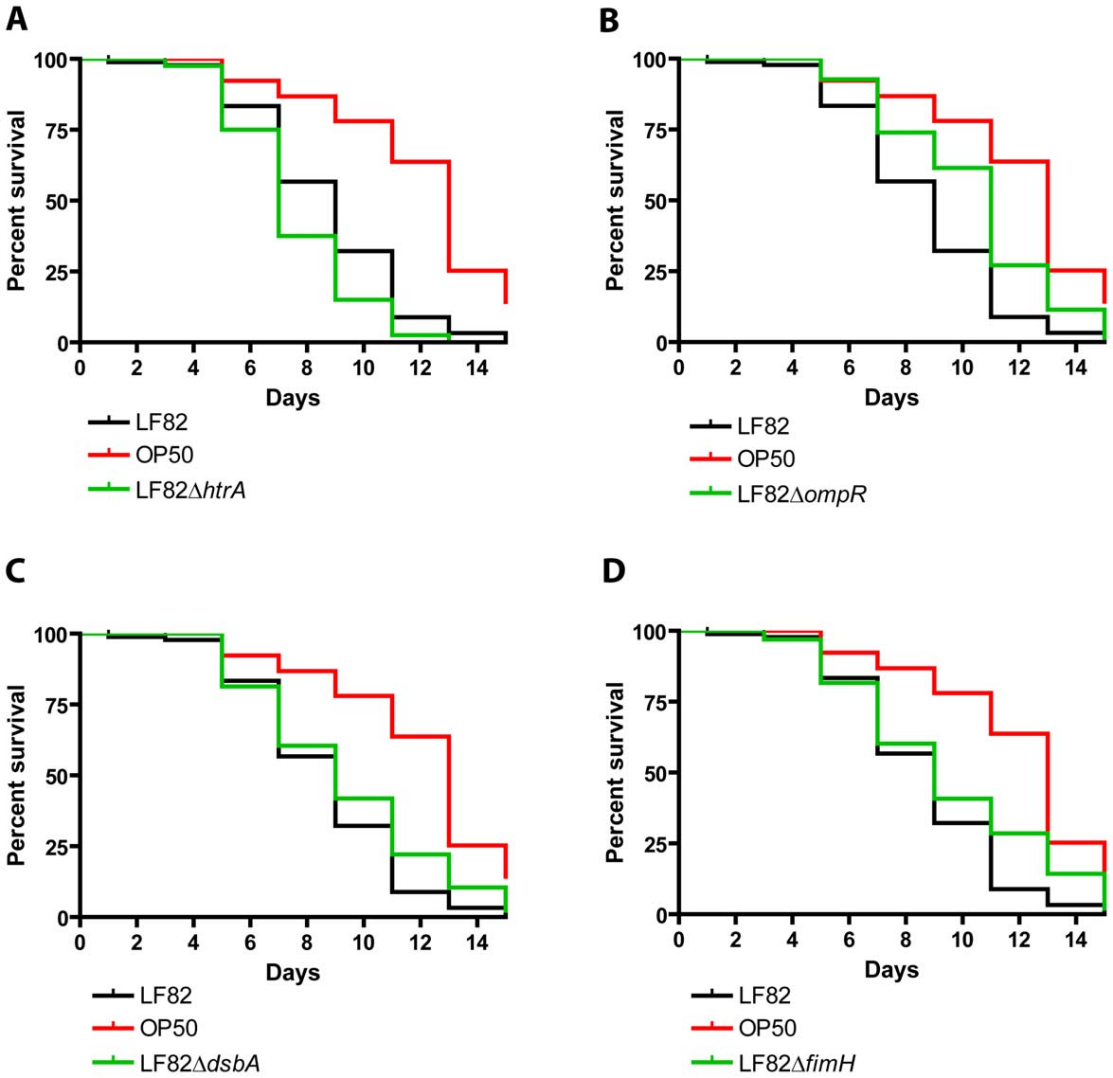
*C. elegans* killing by LF82 is independent of known virulence factors. Nematode killing assays showing that the *C. elegans* killing phenotype does not depend on (**A**) LF82*htrA* (n = 40)(**B**) LF82*ompR* (n = 96) (**C**) LF82*dsbA* (n = 87) or (**D**) LF82*fimH* (n = 84). Nematode survival on the four LF82 mutants is significantly lower than that of nematodes feeding on OP50 (P<0.0001). These virulence factors are known to play a role in pathogenesis in human tissue culture. LF82/pEGFPk (n = 90) and OP50/pEGFPk (n = 91) were included as virulent- and nonvirulent reference strains, respectively.

To gain mechanistic insight into the pathogenesis of LF82 in *C. elegans* we started out by testing LF82 mutants known to display attenuated virulence characteristics in cell culture models. We performed killing assays to compare the life span of nematodes feeding on LF82 derivatives lacking known virulence genes (Figure 3A–D). These include the non-adherent and non-invasive LF82Δ*ompR*, deficient in OmpC expression [19], and LF82Δ*fimH*, deficient in type 1 pilus adhesin expression [15], as well as LF82Δ*htrA*, deficient in intracellular replication [21], and LF82Δ*dsbA* which is incapable of intracellular survival [36]. All four mutants retained the slow-killing phenotype, although the LF82Δ*ompR* mutant displayed attenuated virulence compared to the wild type strain (Figure 3B; P<0.0001). These findings suggested that full pathogenesis of LF82 in the nematode model required additional bacterial factors.

**Figure 4:**
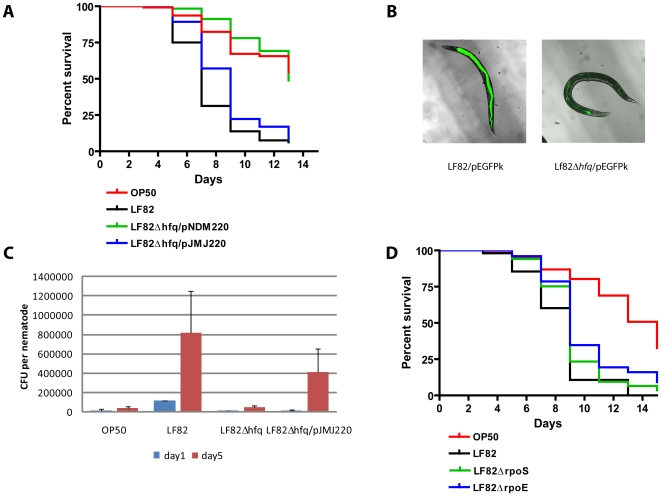
LF82 *hfq* is required for nematode colonization and killing independent of RpoS and RpoE. (**A**) Nematode killing assay demonstrating that LF82-mediated *C. elegans* killing is abolished by deletion of the *hfq* gene. The survival of nematodes feeding on LF82Δ*hfq* containing the empty vector pNDM220 is not significantly different from that of nematodes feeding on OP50 (P = 0.0735). Nematode killing could be restored through complementation by introducing the *hfq* allele on the pJMJ220 plasmid (P<0.0001). (**B**) Nematodes were examined by fluorescence microscopy at day 5 post infection to visualize the infecting bacteria. (**C**) Nematode colonization by LF82 and the *hfq* mutant was measured by CFU determination at days 1 and 5 post infection and compared to that of the non-virulent reference OP50. (**D**) Nematode killing assay showing that LF82-mediated killing of *C. elegans* does not require *rpoS* or *rpoE*, indicating that *hfq* controls virulence expression by a RpoS and RpoE-independent mechanism. Both survival curves are significantly different from the OP50 control (P<0.0001).

Since these mutant strains retained full or partial virulence in the *C. elegans* model, we decided to construct an *hfq* deletion mutant given that Hfq has been shown to play a role in the virulence and general fitness of a number of important bacterial pathogens [37], some of which have been shown to display virulence phenotypes in *C. elegans*. The deletion of the *hfq* gene had a pronounced effect on LF82 virulence in *C. elegans*, virtually abolishing the nematode killing phenotype displayed by the wild type (Figure 4A). The killing rate of nematodes feeding on LF82Δ*hfq* was comparable to that of the control population feeding on OP50. Nematode killing could be restored by re-introducing the *hfq* gene on an IPTG-inducible low-copy-number plasmid pJMJ220, indicating that the observed effect was not due to secondary effects of deleting the chromosomal allele. In order to determine if the attenuated virulence of LF82Δ*hfq* could be caused by inability to colonize the host, we measured the bacterial load in the nematode intestine on days 1 and 5 after infection, respectively (Figure 4C). Whereas LF82 is found in large numbers per nematode, LF82Δ*hfq* is found in low numbers comparable to that of OP50, showing that this mutant is incapable of colonizing the nematode. Nematode colonization could be partially restored by introducing the complementing plasmid pJMJ220. The lack of full complementation is most likely due to insufficient induction due to instability of the inducer. The effect of *hfq* deletion on LF82 colonization was evident in the fluorescence microscope as well. At day 5, GFP-expressing LF82 filled the entire intestinal lumen, whereas the *hfq* mutant gave rise to a much weaker fluorescent trace (Figure 4B).

Given that Hfq plays important roles in orchestrating the function of both the stationary-phase sigma factor RpoS and the envelope stress response sigma factor RpoE, we investigated whether these two genes might be responsible for the observed effect of *hfq* deletion on LF82 virulence. Deletion mutants were constructed and analyzed in the *C. elegans* killing assay. As shown in Figure 4D, the LF82Δ*rpoS* and LF82Δ*rpoE* mutants both displayed killing phenotypes comparable to that of LF82, indicating that Hfq acts independently of the RpoS and RpoE sigma factors in controlling virulence.

### LF82 colonization affects the nematode intestinal cells

**Figure 5:**
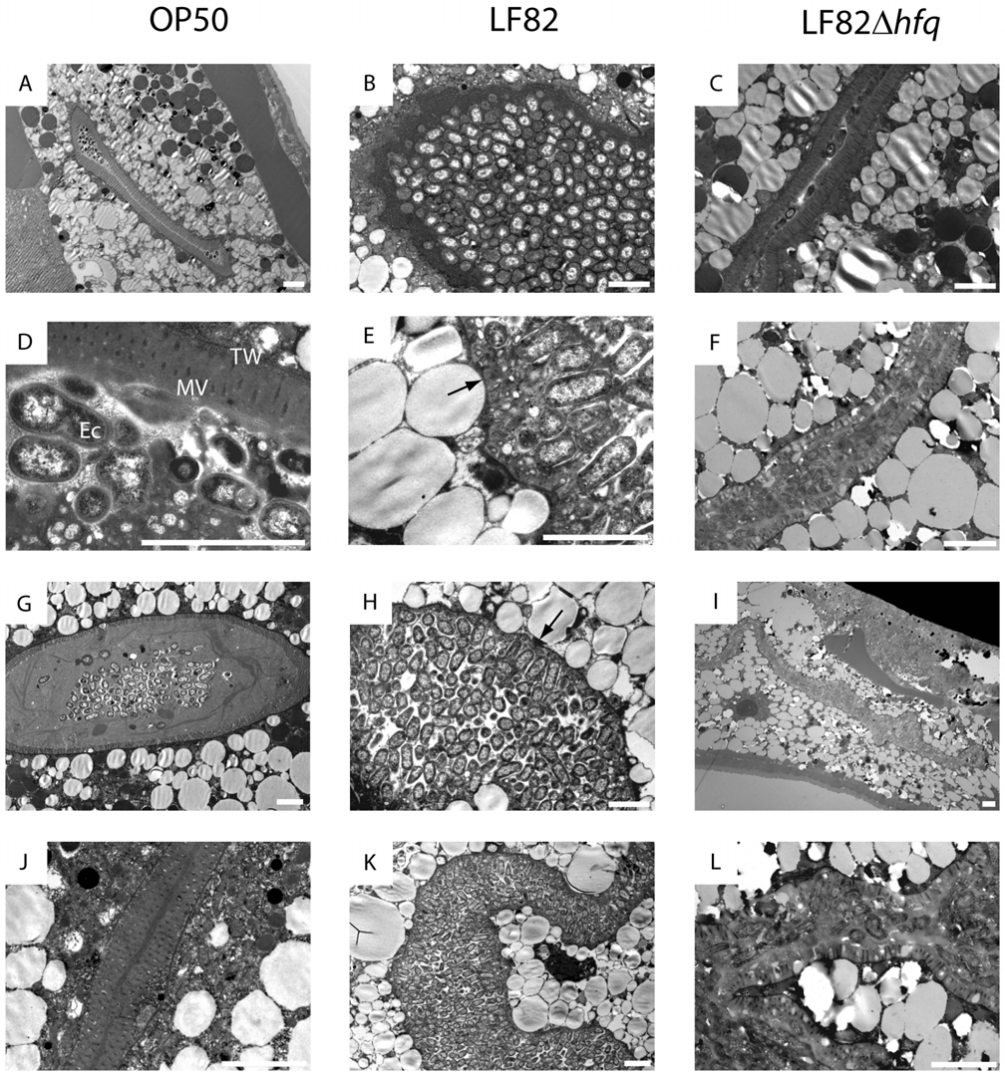
LF82 colonization affects the nematode epithelium. Electron micrographs of *C. elegans* thin sections. (**A–C**) Nematodes feeding for three days on OP50, LF82 and LF82Δ*hfq*, respectively. (**D–L**) Nematodes feeding for five days. MV, microvilli; TW, terminal web; Ec, *E. coli* bacteria. Damaged apical microvilli are indicated by black arrows. Scale bars represent 5 µm.

To further analyze the link between LF82 colonization and host killing described above, we performed thin section electron microscopy of nematodes infected with OP50, LF82 and LF82Δ*hfq*. At day three of the infection a clear difference in the degree of colonization was observed (Figure 5A–C). Whereas OP50 and LF82Δ*hfq* were found in small numbers in the intestinal lumen, LF82 was present in much higher numbers. This observation correlates with CFU determinations and fluorescence microscopic analyses shown in Figure 4. Despite the increased bacterial load, the intestinal barrier of LF82-infected nematodes appeared normal with intact terminal web and apical microvilli (Figure 5B). Changes in the nematode intestinal cell surface became apparent on day five of the infection (Figure 5E, H, K). Although the terminal web appeared to be intact, microvilli were partially or completely effaced (Figure 5E, H; black arrows). The bacteria were oriented randomly with respect to the intestinal surface and did not seem to be directly attached to the epithelium surface. At this stage of the infection, colonization outside the nematode intestine was also observed (not shown). It is not clear, however, whether extraintestinal colonization occurs as a result of host death. In agreement with results of the killing and colonization assays, LF82Δ*hfq* not only colonized the nematodes to a lesser extent but also left the intestinal surface intact throughout (Figure 5I–L).

### LF82 *hfq* is required for host cell invasion and intracellular survival and replication in cultured macrophages

**Figure 6:**
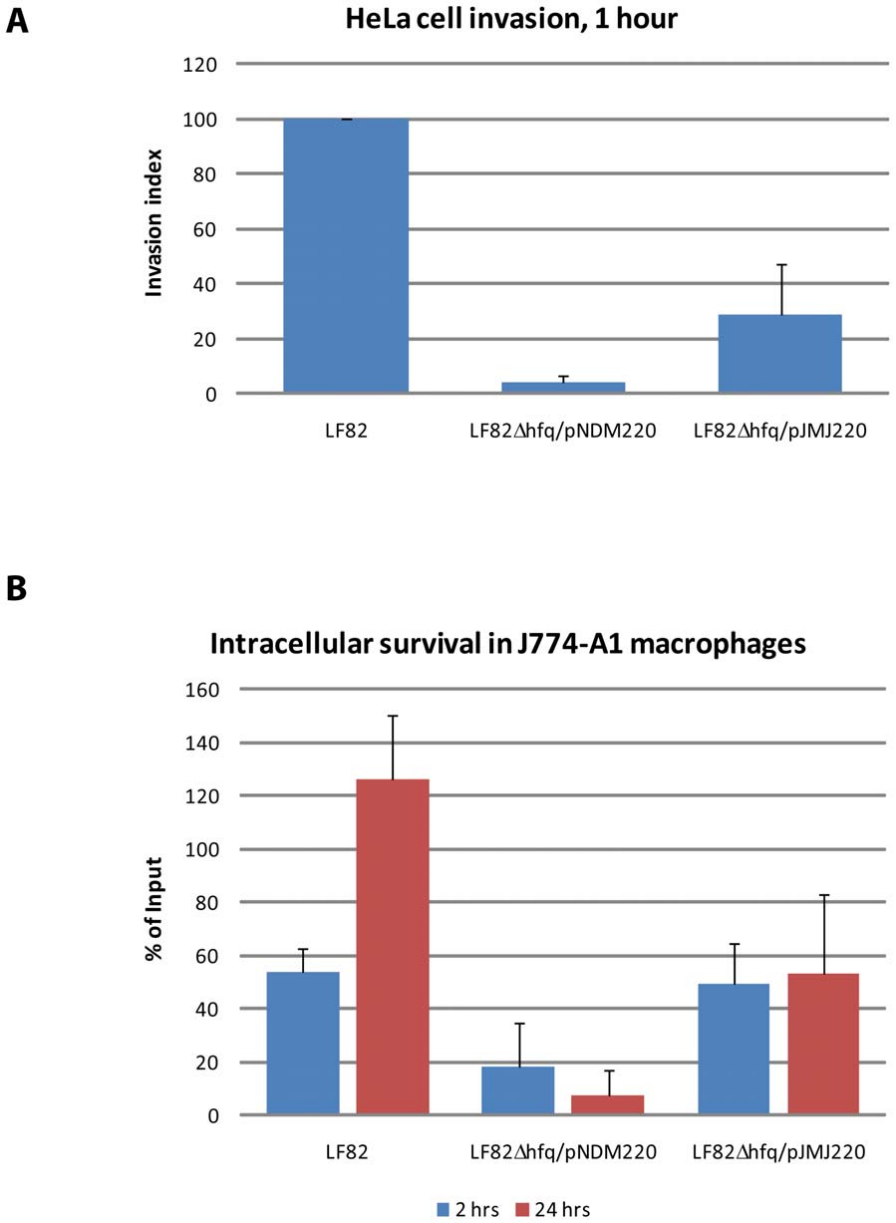
LF82 *hfq* is involved in host cell invasion and intracellular survival in macrophages. (**A**) Effect of LF82 *hfq* deletion on HeLa cell invasion as determined by gentamicin protection assay. The *hfq* deletion mutant contained either the empty vector pNDM220 or the complementing Hfq-expression plasmid pJMJ220. The invasiveness of mutant strains is expressed relative to the number of intracellular CFU determined for wild type LF82 (index 100) 1 hour post infection. (**B**) Intracellular survival and replication of wild type LF82, the *hfq* deletion mutant containing the empty vector LF82Δ*hfq*/pNDM220, and the complemented strain LF82Δ*hfq*/pJMJ220 in J774-A1 macrophage culture determined by gentamicin protection at 2 and 24 hours post infection, respectively.

We performed gentamicin protection assays using cultured HeLaS3 cells and J774-A1 macrophages in order to investigate if deletion of *hfq* would also affect LF82 invasion and intracellular proliferation, respectively. HeLaS3 cell monolayers were infected with LF82, LF82Δ*hfq* and the complementing LF82Δ*hfq*/pJMJ220 strains at a MOI of 100 bacteria per cell and intracellular CFU values were determined after 1 hour. Deletion of *hfq* reduced the invasive index to 4%, indicating that Hfq plays a role during active invasion of epithelial cells (Figure 6A). Introducing the *hfq* allele on a plasmid restored the invasive ability to 29%. The incomplete complementation may be due to insufficient induction of the plasmid encoded Hfq expression system or plasmid instability. Similarly, when analyzed for the ability to survive and replicate inside cultured J774-A1 macrophages, the *hfq* deletion mutant displayed drastically reduced CFU numbers (Figure 6B). Already at 2 hours post infection, the number of living intracellular LF82Δ*hfq* cells (18% of the input) was less than half of that of the wild type and at 24 hours post infection, this number had dropped to 8%. Meanwhile, the wildtype LF82 had increased in numbers in accordance with previous reports [7], [17]. Expressing Hfq from a plasmid was able to restore the ability of the *hfq* mutant to resist phagocytosis. Together, these results show that Hfq is important for two hallmark features of adherent-invasive *Escherichia coli* strains, namely their ability to invade epithelial cells and the capacity to survive and multiply inside macrophages.

### Deletion of LF82 *hfq* affects both stress tolerance and motility

**Figure 7:**
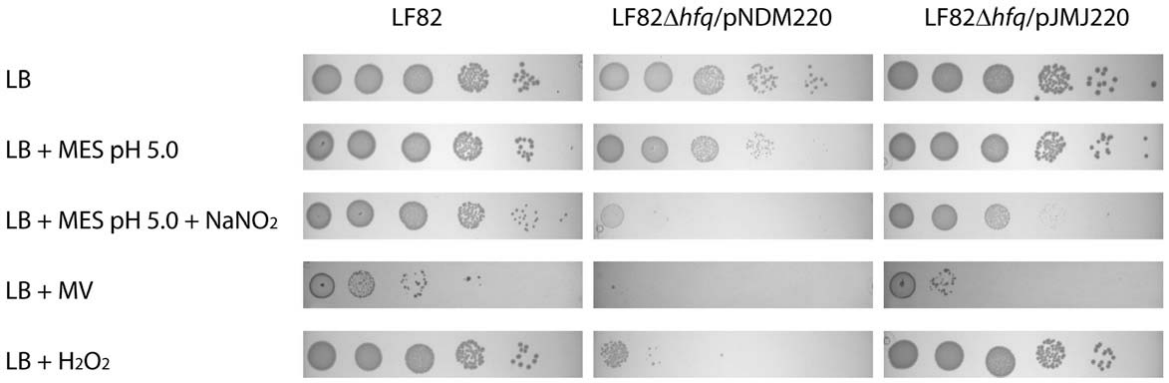
Decreased stress tolerance of the LF82Δ*hfq* mutant. 10-fold serial dilutions were made from exponentially growing cultures and plated, starting with 10^5^ bacterial cells (left), and grown over night at 37°C. Stress tolerance of wild type LF82 was compared to that of LF82Δ*hfq*/pNDM220 and the complemented deletion strain LF82Δ*hfq*/pJMJ220 on plates containing no stress (LB), low pH (100 mM MES pH 5.0), reactive nitrogen species (100 mM MES pH 5.0 + 1 mM NO_2_), and reactive oxygen species (1 mM methyl viologen or 1 mM H_2_O_2_).

As mentioned, a distinguishing feature of adherent-invasive LF82 bacteria is their ability to survive and replicate inside macrophages without inducing cell death [38] or escaping from the endocytic pathway [20]. By contrast to other intracellular bacterial pathogens, which evade phagocytosis by either escaping the endosome or preventing its fusion with the lysosomal compartment, LF82 is able to replicate within the acidic and cathepsin D-positive vacuolar environment of mature phagolysosomes [20]. As the reduced ability of the *hfq* mutant to resist phagocytosis might reflect a low tolerance to acidic conditions and reactive oxygen and nitrogen species characteristic of the mature phagolysosome, we tested the ability of the LF82Δ*hfq* mutant to grow on agar plates containing various chemical stress conditions. As shown in Figure 7, LF82Δ*hfq* growth was only slightly impaired on LB agar plates. Acidification by addition of 100 mM MES pH 5.0 made the growth impairment of the *hfq* mutant more pronounced. Inclusion of reactive nitrogen species in the form of 1 mM acidified sodium nitrite severely impeded growth of the *hfq* mutant while leaving the wild type unaffected. Reactive oxygen species formed by addition of 1 mM methyl viologen or 1 mM hydrogen peroxide had an equally toxic effect on the *hfq* mutant, demonstrating that Hfq is important for the ability of LF82 to resist a variety of chemical stress conditions. In all cases, bacterial growth could be restored to nearly wild type levels by introducing the complementing plasmid pJMJ220.

In addition to reactive oxygen and nitrogen species most multicellular organisms including *C. elegans* produce an array of antimicrobial peptides (AMPs) as a first line of defense against invading pathogens. In general, the AMPs can be classified into three broad groups [39]: α-helical peptides (e.g., LL37, novicidin, magainin and cecropin A); β-sheet peptides stabilized by one or more disulfide bridges (e.g., hBD2, HD5 and iseganan) or extended structures enriched in particular amino acids, such as proline and tryptophan (e.g., the indolicidin derivative omiganan). One possible reason for the observed reduction in virulence of the *hfq* strain may be that it has become more sensitive to AMPs encoded by the nematode host. We measured the sensitivity of the wild type, Δ*hfq*, and *hfq-*complementing strains against a panel of AMPs representing the predominant classes of host defense peptides described above (Table 1). Apparently, no difference in sensitivity against any of the AMPs tested here was observed between the wild type and the *hfq* strain. This result is in contrast to a previous study in which deletion of *hfq* in uropathogenic *E. coli* was found to greatly increase bacterial sensitivity to the antimicrobial peptide polymyxin B [40].

**Table 1:** Sensitivity to antimicrobial compounds (MIC in µg/ml).

Antimicrobialcompound	LF82*	LF82*Δ*hfq*	LF82*Δ*hfq*/pNDM220-*hfq*
Cathelicidin LL37	1	1	2
Novicidin	0.5	0.5–1	1
Iseganan	1	0.5	1–2
Omiganan	0.5 – 1	0.5	1
Magainin I	1	2	2
Polymyxin B	0.125	0.125	0.25
Cecropin	32	32	32
hBD2	16	8	16
HD5	2	2–4	2–4
Lysozyme	8	8	8

**Figure 8:**
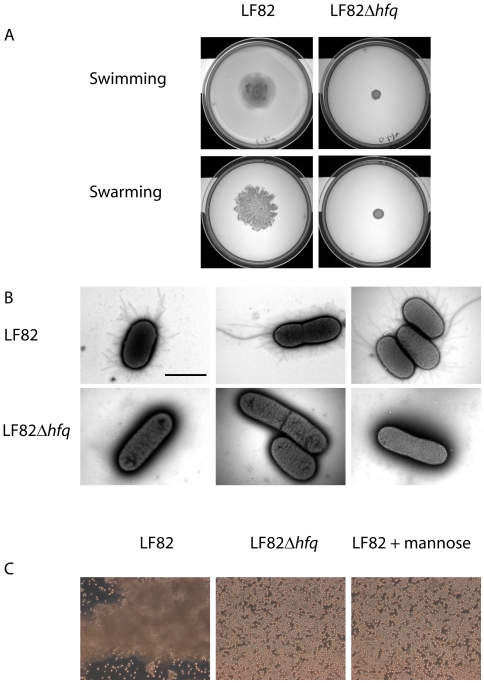
Motility defects in the LF82Δ*hfq* mutant. Growth of LF82 and LF82Δ*hfq* on (**A**) swimming motility agar (0.3% Difco Agar; 0.4% glucose in LB) or swarming motility agar (0.5% Eiken agar; 0.4% glucose in LB). Images were taken after 8 and 18 hours of growth at 37°C, respectively. (**B**) Negative-stain electron microscopy showing that the LF82Δ*hfq* mutant is devoid of both Type 1 pili (black arrow) and flagellae (white arrow). Black scale bar represents 2 µm (**C**) Yeast agglutination assay showing LF82*hfq*-dependent agglutination of yeast cells in suspension. The prevention of agglutination by mannose addition suggests that LF82 binding of yeast cells is mediated by Type 1 pili.

Previous studies have demonstrated the importance of Hfq for bacterial motility [40]–[42]. Using swimming and swarming motility agar plates we investigated the role of LF82 Hfq on motility. Figure 8A shows that the *hfq* deletion mutant was deficient in both swimming and swarming motility. We examined the bacteria using negative-stain electron microscopy in order to determine if the observed defects in cell adhesion and motility could be explained by lack of Type I pili and flagellae, respectively. The electron micrographs shown in Figure 8B reveal that LF82Δ*hfq* mutant cells lack both pili and flagellae. In order to confirm the lack of type I pili, we performed a yeast agglutination assay shown in Figure 8C. Whereas wild type LF82 was capable of agglutinating yeast cells, the *hfq* mutant did not induce any detectable yeast cell agglutination. Yeast agglutination could be blocked by inclusion of 3% mannose, consistent with type I pili-dependent agglutination.

## Discussion

Most of the current knowledge regarding the mechanism of AIEC pathogenesis is derived from studies of the prototype strain LF82 infecting various cell cultures. In order to expand our knowledge of AIEC pathogenesis by identifying additional virulence determinants we set out to determine if the nematode *C. elegans* could be used as a small animal model for LF82 infection. Despite having a simpler immune system consisting of innate mechanisms only, many antimicrobial proteins produced by *C. elegans* in response to bacterial infection are also conserved in higher organisms [43], and this infection model may hence provide an orthogonal approach to the study of AIEC infection. We found that LF82 was capable of mounting a persistent colonization of the nematode intestine which led to a slow-killing phenotype over the course of several days. By measuring the intestinal LF82 load subsequent to removal of the nematodes from LF82-containing agar plates (Figure 2B) we demonstrate that LF82 bacteria are capable of resisting clearance by peristalsis and even capable of increasing in number as the infection progressed. The nematode killing assays displayed a consistent lag phase of at least three days before the worm killing phenotype was manifested, suggesting that intestinal colonization is a prerequisite of pathogenicity in *C. elegans*. The fact that worms that were transferred to a non-virulent *E. coli* substrate after 24 hours of LF82 exposure displayed a delayed killing phenotype (Figure 2C) is consistent herewith. Thus, LF82 pathogenesis in *C. elegans* as in mammalian hosts relies on bacterial colonization.

In human cell culture, LF82 adheres to the brush border of the ileal epithelium via specific interactions between type 1 pili on the bacterial surface and carcinoembryonic antigen-related cell adhesion molecules (CEACAM6) on the apical surface of ileal epithelial cells [15]. In a recent study, LF82 was shown to colonize the intestinal mucosa and to induce inflammation and severe colitis in transgenic mice expressing human CEACAMs [24]. Colonization of the transgenic mice was also dependent on bacterial type 1 pili expression, demonstrating the specificity of the molecular interactions mediating LF82 adherence. To our knowledge, CEACAM proteins have not been found in *C. elegans*, and BLAST searches using human CEACAM6 against the nematode genome did not reveal any orthologous sequences, so LF82 colonization must rely on a different set of host-pathogen interactions. Our finding that an LF82 *fimH* deletion mutant, which is unable to express the type 1 pili adhesin, did not show reduced worm killing, is consistent with this notion. Having established the killing phenotype in *C. elegans*, we went on to examine if genes that were found previously to play important roles for LF82 survival and intracellular multiplication in cell culture would be equally important for virulence in the nematode model system. Importantly, LF82 mutants with deletions of *htrA*, *dsbA*, *or fimH* displayed no attenuation of virulence in *C. elegans*, indicating that nematode killing relies on mechanisms that are distinct from those involved in cell invasion and resistance to phagocytosis. This is expected since LF82 does not appear to invade the intestinal cells in *C. elegans* and the nematode scavenger cells (coelomocytes) are not believed to be involved in phagocytosis. Mutation of the LF82 *ompR* gene resulted in an intermediary killing phenotype (Figure 3B). Although this mutant behaves different from the non-virulent control (OP50) it is also significantly altered compared to the wild-type pathogenic strain, so we conclude that OmpR may play a minor role in the nematode model. The fact that virulence factors known to be highly important in other hosts seem dispensable in *C. elegans* has been reported for other pathogenic bacteria as well. The *plcS* gene of *P. aeruginosa*, which encodes a hemolytic phospholipase, and the *ssaV* gene in the *Salmonella* pathogenicity island 2 were both required for full virulence in the mouse, but did not cause any attenuation of virulence in *C. elegans* [27], [28]. But despite the differences in the mechanism of pathogenicity between *C. elegans* and other model organisms, the worm has proven to be an extremely useful model, since genes not previously known to be involved in virulence of *P. aeruginosa* was identified in *C. elegans* and proven to also cause attenuated virulence in a mouse model [27], [44].

In order to address in more detail the mechanism of AIEC pathogenesis in *C. elegans* we chose to delete the LF82 *hfq* gene, given the fact that the Hfq protein has been shown to play an important role in controlling the virulence of a wide range of pathogenic bacteria [37]. Hfq is a small abundant RNA-binding protein that functions as a global posttranscriptional regulator of gene expression by binding to small regulatory RNA molecules (sRNAs) and facilitating their interaction with mRNA molecules [45]. The pleiotropic nature of the *hfq* gene is illustrated by a recent study reporting that *hfq* deletion affects the expression of hundreds of genes both at the mRNA and protein level [46]. Our results presented in Figure 4 shows that deletion of LF82*hfq* has a profound effect on *C. elegans* colonization and killing, virtually abolishing the virulence potential. The survival of worms feeding on LF82Δ*hfq* was comparable to those feeding on the avirulent reference strain OP50 (Figure 4A) and virulence attenuation was associated with loss of colonization potential as determined by CFU measurement and fluorescence microscopy (Figure 4B, C). Hfq functions indirectly in control of bacterial stress responses by binding to sRNA molecules and mediating their regulatory function. Some of these sRNAs control the activity of master regulators of bacterial stress response, the sigma factors RpoS and RpoE. RpoS, which is also known as the stationary-phase sigma factor, regulates the expression of a number of genes promoting bacterial adaptation to various external stresses, including starvation, UV radiation, osmotic stress, elevated temperature, low pH and oxidative stress [47]. The envelope stress sigma factor RpoE is activated in response to extracytoplasmic stresses that produce misfolded proteins in the outer membrane and hence plays an important role in maintaining cell envelope integrity [48]. We find that virulence attenuation in the LF82Δ*hfq* mutant is independent of *rpoS* and *rpoE* (Figure 4D), demonstrating that Hfq acts to control worm colonization and killing by a different, yet uncharacterized, genetic pathway.

Although LF82 infection in *C. elegans* appears to rely on molecular mechanisms other than those identified *in vitro* using cultured cells, we reasoned that the *hfq* gene might still play a role during infection of cell culture. We tested the ability of LF82Δ*hfq* to invade epithelial cells and resist phagocytosis in cultured macrophages by performing gentamycin protection assay monitoring the number of bacteria entering cell monolayers. The results shown in Figure 6A show that the invasive ability of the *hfq* mutant was severely reduced. We observed a consistent drop in the invasiveness of this mutant to about 4% of wild type LF82 levels. The absolute number of CFU recorded was low (in the order of 0.1% of bacterial input) probably reflecting the fact that these cells express little or no CEACAM6 protein on their surface [15]. Similarly, the ability of LF82Δ*hfq* to resist phagocytosis by cultured macrophages was significantly reduced (Figure 6B). A clear reduction in CFU recovery was apparent already after 2 hours of incubation and this difference became even more pronounced after 24 hour of incubation. These results demonstrate that LF82 Hfq plays an important role in promoting bacterial adaptation to the multiple stress conditions encountered in the phagocytic vacuole. The importance of *hfq* in stress response was further confirmed by the stress tolerance assay presented in Figure 7. We also analyzed the ability of LF82 and LF82Δ*hfq* to resist the addition of AMPs. Considering the effect of *hfq* deletion on *C. elegans* killing we were surprised to find that the two strains were equally tolerant toward a wide range of antimicrobial peptides (Table 1). A possible explanation for the lack of AMP hypersensitivity may be that none of the tested AMPs are expressed naturally in *C. elegans* since e.g. the worm lysozymes are quite different from the chicken-type lysozyme tested here [49]. Deletion of *hfq* in uropathogenic *E. coli* has been reported to result in hypersensitivity towards the AMP polymyxin B, in particular at elevated concentrations (5 µg/ml) [40]. Thus, we cannot exclude the possibility that LF82 *hfq* confers resistance against AMPs expressed as part of the *C. elegans* innate immune defense.

Previous studies in *Salmonella enterica* Typhimurium [41], *Pseudomonas aeruginosa* [50] and uropathogenic *Escherichia coli* [40] have established a role of Hfq in bacterial motility. Since bacterial flagella-mediated motility has been shown to accelerate *Salmonella* invasion of cultured cells [51], we investigated if the non-invasive LF82Δ*hfq* had motility defects. As shown in Figure 8A, LF82Δ*hfq* was incapable of both swimming and swarming motility. Defects in cell invasion and motility could be explained by the lack of surface structures, as shown in Figure 8B. Interestingly, the LF82Δ*dsbA* and LF82Δ*ompR* mutants, both of which retained virulence in *C. elegans* (Figure 3), have been reported to lack flagella and type I pili [19], [22]. Thus, the effect of *hfq* deletion is not likely to result from reduced expression of flagellae or type I pili alone. The results presented here add further to the complexity of AIEC virulence by demonstrating that LF82 can colonize and kill *C. elegans* by a mechanism which is distinct from those described previously. We find that LF82 virulence in the nematode infection model is strictly dependent on the function of the Hfq RNA chaperone. The fact that this finding in *C. elegans* also proved relevant in the mammalian cell culture system demonstrates that *C. elegans* can function as a useful host model system to study the pathogenesis of LF82. These important findings warrant new experiments to identify both the sRNAs involved in Hfq mediated control of virulence and their corresponding mRNAs targets.

## Supporting Information

Table S1 **Bacterial and nematode strains, plasmids and primers used for this study.**
(DOC)
