# Supplementary material for: A Role for the RNA Chaperone Hfq in Controlling Adherent-Invasive Escherichia coli Colonization and Virulence
Source: PLoS One. 2011 Jan 26;6(1):e16387. doi: 10.1371/journal.pone.0016387 (PMC3027648; doi:10.1371/journal.pone.0016387)
Supplement: Table S1 — Bacterial and nematode strains, plasmids and primers used for this study. (DOC) [file pone.0016387.s001.doc]

**Table S1: Bacterial and nematode strains, plasmids and primers used for this study**

| **Name** | **Description** | **Reference** |
| --- | --- | --- |
| **Bacterial strains** |  |  |
| OP50 | Uracil auxotrophic *E. coli* B | [1] |
| LF82 | Prototypical AIEC isolate; serotype O83:H1; | [2] |
| LF82* | Isogenic LF82;  *ampC* | A. Darfeuille-Michaud |
| LF82*Δ*hfq* | Isogenic LF82* with *hfq* deletion; Cm^R^ | This study |
| LF82*Δ*rpoS* | Isogenic LF82* with *rpoS* deletion; Kan^R^ | This study |
| LF82*Δ*rpoE* | Isogenic LF82* with *rpoE* deletion; Cm^R^ | This study |
| LF82Δ*htrA* | Isogenic LF82 with *htrA* deletion; Kan^R^ | [3] |
| LF82Δ*dsbA* | Isogenic LF82 with *dsbA* deletion; Kan^R^ | [4] |
| LF82Δ*ompR* | Isogenic LF82 with *ompR* deletion; Kan^R^ | [5] |
| LF82Δ*fimh* | Isogenic LF82 with *fimH* deletion; Kan^R^ | [6] |
| BW25113 | BW25113 (*lacI*^q^ *rrnB*_T14_ Δ*lacZ*_WJ16_ *hsdR514* Δ*araBAD*_AH33_ Δ*rhaBAD*_LD78_) | [7] |
| BW25113Δ*rpoS* | Isogenic BW25113 with *rpoS* deletion; Kan^R^ | Poul Valentin-Hansen |
| BW25113Δ*rpoE* | Isogenic BW25113 with *rpoE* deletion; Cm^R^ | [8] |
| DH5α | F^-^ 080d*lacZ*ΔM15 Δ(*lacZYA-argF*)*U169 deoR recA1 endA1 hsdR17*(r_K_^-^ m_K_^+^) *phoA supE44* 1- *thi*-*1 gyrA96 relA1* | Promega |
| ***C. elegans* strain** |  |  |
| *glp-4* | glp-4(bn2ts) temperature-sensitive germ-line mutant; sterile at 25°C; obtained from the *Caenorhabditis* Genetics Stock Center | **[9]** |
| **Plasmids** |  |  |
| pKD46 | pBAD cloning vector harboring the Red recombinase genes from from λ phage; Amp^R^; GenBank^TM^ Accession number AY048746 | [7] |
| pKD3 | Template plasmid for λ Red mediated gene disruption; Cm^R^; GenBank^TM^ Accession number AY048742 | [7] |
| pKD4 | Template plasmid for λ Red mediated gene disruption; Kan^R^; GenBank^TM^ Accession number AY048743 | [7] |
| pNDM220 | R1 derivative; Amp^R^; *lacI*^q^; P_A1/O4/O3_ MCS | [10] |
| pJMJ220 | IPTG-inducible Hfq expression plasmid | This study |
| pACYC177 | *aphA* gene source | New England Biolabs |
| pEGFPk | pEGFP derivative; Kan^R^ | This study |
| pQW58 | pUC19 derivative; mCherry gene fusion vector | Qing Wang |
| **Primers** |  |  |
| JMJ63 | 5’-ATGGCTAAGGGGCAATCTTTACAAGATCCGTTCCTGAAgtgtaggctggagctgcttcg | This study |
| JMJ64 | 5’-TTATTCGGTTTCTTCGCTGTCCTGTTGCGCGGAAGCATATGAATATCCTCCTTAGTTCC | This study |
| JMJ153 | 5’-CCCCCTCGAGAAGGAAAAGAGAGAATGGCTAAGG | This study |
| JMJ154 | 5’-CCCCCGAATTCTTATTCGGTTTCTTCGCTGTCC | This study |
|  |  |  |

Reference List

1. Brenner S (1974) The genetics of Caenorhabditis elegans. Genetics 77: 71-94.

2. Darfeuille-Michaud A, Neut C, Barnich N, Lederman E, Di MP, et al. (1998) Presence of adherent Escherichia coli strains in ileal mucosa of patients with Crohn's disease. Gastroenterology 115: 1405-1413.

3. Bringer MA, Barnich N, Glasser AL, Bardot O, Darfeuille-Michaud A (2005) HtrA stress protein is involved in intramacrophagic replication of adherent and invasive Escherichia coli strain LF82 isolated from a patient with Crohn's disease. Infect Immun 73: 712-721.

4. Bringer MA, Rolhion N, Glasser AL, Darfeuille-Michaud A (2007) The oxidoreductase DsbA plays a key role in the ability of the Crohn's disease-associated adherent-invasive Escherichia coli strain LF82 to resist macrophage killing. J Bacteriol 189: 4860-4871.

5. Rolhion N, Carvalho FA, Darfeuille-Michaud A (2007) OmpC and the sigma(E) regulatory pathway are involved in adhesion and invasion of the Crohn's disease-associated Escherichia coli strain LF82. Mol Microbiol 63: 1684-1700.

6. Barnich N, Carvalho FA, Glasser AL, Darcha C, Jantscheff P, et al. (2007) CEACAM6 acts as a receptor for adherent-invasive E. coli, supporting ileal mucosa colonization in Crohn disease. J Clin Invest 117: 1566-1574.

7. Datsenko KA, Wanner BL (2000) One-step inactivation of chromosomal genes in Escherichia coli K-12 using PCR products. Proc Natl Acad Sci U S A 97: 6640-6645.

8. Johansen J, Rasmussen AA, Overgaard M, Valentin-Hansen P (2006) Conserved small non-coding RNAs that belong to the sigmaE regulon: role in down-regulation of outer membrane proteins. J Mol Biol 364: 1-8.

9. Beanan MJ, Strome S (1992) Characterization of a germ-line proliferation mutation in C. elegans. Development 116: 755-766.

10. Gotfredsen M, Gerdes K (1998) The Escherichia coli relBE genes belong to a new toxin-antitoxin gene family. Mol Microbiol 29: 1065-1076.
